# Supplementary material for: A Methodological Framework for Assessing the Benefit of SARS-CoV-2 Vaccination following Previous Infection: Case Study of Five- to Eleven-Year-Olds
Source: Vaccines (Basel). 2023 May 16;11(5):988. doi: 10.3390/vaccines11050988 (PMC10220644; doi:10.3390/vaccines11050988)
Supplement: Supplementary file 1 [file vaccines-11-00988-s001.zip › vaccines-2374887-supplementary.pdf]

vaccines MDPI

Supplementary Materials

Parameterising the framework for hospital admissions averted

Previous work led by author Brown undertook detailed analysis of all child hospital admissions with COVID-19 in England, conducted as part of the British Heart Foundation Data Science Centre's COVID-IMPACT consortium [1].

This analysis provided high quality data on the number of overall hospital admissions where SARS-CoV-2 was a contributing factor in children aged five to 11 associated with their first confirmed infection from July 2020 up to the end of February 2022, separated out by whether a child had evidence of an underlying health condition (UHC). For this estimate, a UHC was an underlying health condition recognised as placing the child at potentially having higher risk of severe disease in the UK Green Book [2]. The analysis also provided estimates of the number of children with and without an UHC in the overall population. For details of the methods in that study please see Wilde et al. (under review) [3].

The UK Office for National Statistics Schools Infection Survey reported that 82% of primary school pupils in England tested positive for SARS-CoV-2 antibodies by March 2022, and an incidence of 98% from mid December 2021 to mid June 2022 among 2 to 11 year olds, denoting high levels of exposure to SARS-CoV-2 as vaccination roll out did not begin until April 2022 [4,5].

Estimates for the range of protection provided against hospitalisation provided by previous infection alone and previous infection plus vaccination are taken from Lin et al. and Bobrovitz et al. [6,7] and assumed the same for children with and without UHC.

For estimating the bounds on a linear waning rate for protection against hospitalisation on reinfection, we used a minimum and maximum waning rate of 0.6 and 1.7 percentage points each month informed by the fits shown in Figure A1 below.

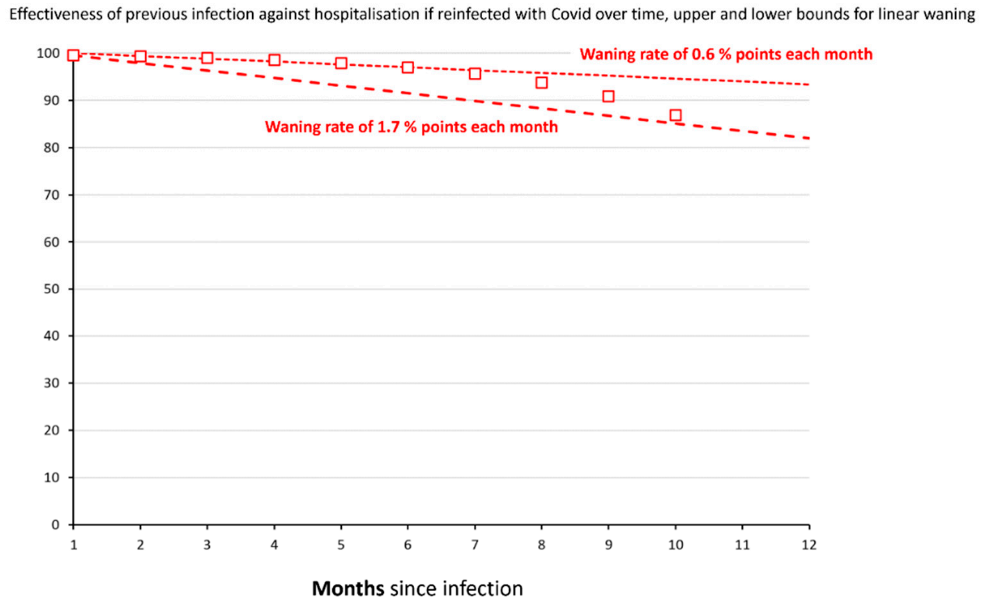

Figure S1. – fits of maximum and minimum linear waning to protection of previous infection against hospitalisation on new infection.

Parameterising the framework for Long Covid cases averted

For this analysis, we consider children as a whole group since reliable estimates for parameterising by underlying health condition do not exist to our knowledge.

There exist a wide range estimates of the incidence of Long Covid in children from 1.8% to 14% [8–10], using different cohorts, different definitions of Long Covid and different analysis methods. Three recent studies, all with some sort of control group (albeit with remaining significant limitations), put the percentage of children experiencing symptoms longer than two to three months (different studies use different definitions) following a SARS-CoV-2 infection at between 1.6% and 5% [11–13]. Recent guidance (September 2022) from the American Academy of Pediatrics gives a range of 2-5% of children experiencing ongoing symptoms after 3 months [14]. We use a central estimate of 3.5%, giving an N_A of 200,000 children in England (out of 5.7 million) who would have experienced Long Covid if all had been infected for the first time.

Vaccination and previous infection can reduce new incidence of Long Covid in two ways: firstly by preventing reinfection in the first place and secondly by reducing the chance of developing Long Covid once infected [15]. If z is the effectiveness in preventing infection and v the effectiveness of preventing Long Covid once infected, then the overall effectiveness, w, in preventing Long Covid is given by: $w=1-(1-z)(1-v)$, where z and v can vary for protection from infection or hybrid immunity.

Estimates for the range of protection provided against reinfection provided by previous infection along and previous infection and vaccination are taken from Bobrovitz et al. [7] and assumed the same for children with and without the specified underlying health conditions.

References

1. Wood, A.; Denholm, R.; Hollings, S.; Cooper, J.; Ip, S.; Walker, V.; Denaxas, S.; Akbari, A.; Banerjee, A.; Whiteley, W.; et al. Linked Electronic Health Records for Research on a Nationwide Cohort of More than 54 Million People in England: Data Resource. *BMJ* **2021**, *373*, n826, doi:10.1136/bmj.n826.
2. COVID-19: The Green Book, Chapter 14a Available online: https://www.gov.uk/government/publications/covid-19-the-green-book-chapter-14a (accessed on 27 February 2023).
3. Wilde, H.; Tomlinson, C.; Mateen, B.; Selby, D.; Kanthimathinathan, H.K.; Ramnarayan, P.; Pré, P. du; Johnson, M.; Pathan, N.; Gonzalez-Izquierdo, A.; et al. Hospital Admissions Linked to SARS-CoV-2 Infection in Children: A Cohort Study of 3.2 Million First Ascertained Infections in England. *BMJ : British Medical Journal* **2023**.
4. COVID-19 Schools Infection Survey, England: Pupil Antibody Data, January to February 2022 - Office for National Statistics Available online: https://www.ons.gov.uk/releases/covid19schoolsinfectionsurveyenglandpupilantibodydatajanuarytofebruary2022 (accessed on 1 August 2022).
5. Coronavirus (COVID-19) Infection Survey Technical Article: Cumulative Incidence of the Percentage of People Who Have Been Infected with COVID-19 by Variant and Age, England - Office for National Statistics Available online: https://www.ons.gov.uk/peoplepopulationandcommunity/healthandsocialcare/conditionsanddiseases/articles/coronavirusco-vid19infectionsurveytechnicalarticlecumulativeincidenceofthenumber-ofpeoplewhohavebeeninfectedwithcovid19byvariantandageengland/9february2023#data-sources-and-quality (accessed on 11 February 2023).
6. Lin, D.-Y.; Gu, Y.; Xu, Y.; Zeng, D.; Wheeler, B.; Young, H.; Sunny, S.K.; Moore, Z. Effects of Vaccination and Previous Infection on Omicron Infections in Children. *New England Journal of Medicine* **2022**, *0*, null, doi:10.1056/NEJMc2209371.
7. Bobrovitz, N.; Ware, H.; Ma, X.; Li, Z.; Hosseini, R.; Cao, C.; Selemon, A.; Whelan, M.; Premji, Z.; Issa, H.; et al. Protective Effectiveness of Previous SARS-CoV-2 Infection and Hybrid Immunity against the Omicron Variant and Severe Disease: A Systematic Review and Meta-Regression. *The Lancet Infectious Diseases* **2023**, *0*, doi:10.1016/S1473-3099(22)00801-5.
8. Stephenson, T.; Stephenson, T.; Pereira, S.P.; Shafran, R.; De Stavola, B.; Rojas, N.; McOwat, K.; Simmons, R.; Zavala, M.; O'Mahoney, L.; et al. *Long COVID - the Physical and Mental Health of Children and Non-Hospitalised Young People 3 Months after SARS-CoV-2 Infection; a National Matched Cohort Study (The CLoCk) Study.*; In Review, 2021;
9. Molteni, E.; Sudre, C.H.; Canas, L.S.; Bhopal, S.S.; Hughes, R.C.; Antonelli, M.; Murray, B.; Kläser, K.; Kerfoot, E.; Chen, L.; et al. Illness Duration and Symptom Profile in Symptomatic UK School-Aged Children Tested for SARS-CoV-2. *The Lancet Child & Adolescent Health* **2021**, *5*, 708–718, doi:10.1016/S2352-4642(21)00198-X.

10. Gurdasani, D.; Akrami, A.; Bradley, V.C.; Costello, A.; Greenhalgh, T.; Flaxman, S.; McKee, M.; Michie, S.; Pagel, C.; Rasmussen, S.; et al. Long COVID in Children. *The Lancet Child & Adolescent Health* **2022**, *6*, e2, doi:10.1016/S2352-4642(21)00342-4.
11. Rao, S.; Lee, G.M.; Razzaghi, H.; Lorman, V.; Mejias, A.; Pajor, N.M.; Thacker, D.; Webb, R.; Dickinson, K.; Bailey, L.C.; et al. Clinical Features and Burden of Postacute Sequelae of SARS-CoV-2 Infection in Children and Adolescents. *JAMA Pediatrics* **2022**, *176*, 1000–1009, doi:10.1001/jamapediatrics.2022.2800.
12. Kikkenborg Berg, S.; Palm, P.; Nygaard, U.; Bundgaard, H.; Petersen, M.N.S.; Rosenkilde, S.; Thorsted, A.B.; Ersbøll, A.K.; Thygesen, L.C.; Nielsen, S.D.; et al. Long COVID Symptoms in SARS-CoV-2-Positive Children Aged 0–14 Years and Matched Controls in Denmark (LongCOVIDKidsDK): A National, Cross-Sectional Study. *The Lancet Child & Adolescent Health* **2022**, *6*, 614–623, doi:10.1016/S2352-4642(22)00154-7.
13. Funk, A.L.; Kuppermann, N.; Florin, T.A.; Tancredi, D.J.; Xie, J.; Kim, K.; Finkelstein, Y.; Neuman, M.I.; Salvadori, M.I.; Yock-Corrales, A.; et al. Post–COVID-19 Conditions Among Children 90 Days After SARS-CoV-2 Infection. *JAMA Network Open* **2022**, *5*, e2223253, doi:10.1001/jamanetworkopen.2022.23253.
14. Post-COVID-19 Conditions in Children and Adolescents Available online: http://www.aap.org/en/pages/2019-novel-coronavirus-covid-19-infections/clinical-guidance/post-covid-19-conditions-in-children-and-adolescents/ (accessed on 3 October 2022).
15. COVID-19 Vaccination Protects against Long COVID Available online: https://www.gavi.org/vaccineswork/covid-19-vaccination-protects-against-long-covid (accessed on 25 October 2022).
